# Fibroblasts in cancer dormancy: foe or friend?

**DOI:** 10.1186/s12935-021-01883-2

**Published:** 2021-03-26

**Authors:** Li Dai, Mao Li, Wei-long Zhang, Ya-Jie Tang, Ya-ling Tang, Xin-hua Liang

**Affiliations:** 1 State Key Laboratory of Oral Diseases and National Clinical Research Center for Oral Diseases, West China Hospital of Stomatology, Sichuan University, Chengdu, China; 2 State Key Laboratory of Microbial Technology, Shandong University, Qingdao, 266237 China

**Keywords:** Fibroblast, Cancer dormancy, CAF, Senescence, Cancer therapy

## Abstract

Cancer dormancy is defined that the residual cancer cells could enter into a state of quiescence and patients remain asymptomatic for years or even decades after anti-tumor therapies. Fibroblasts, which represent a predominant cell type in tumor microenvironment, play a pivotal role in determining the ultimate fate of tumor cells. This review recapitulates the pleiotropic roles of fibroblasts which are divided into normal, senescent, cancer-associated fibroblasts (CAFs) and circulation CAFs in tumor dormancy, relapse, metastasis and resistance to therapy to help the treatment of cancer metastasis.

## Background

Cancer dormancy is defined as a latency period that cancer becomes indolent and remains in an undetectable state for years before emerging as an overtly proliferative disease [1]. This latency can be regarded as a quiet recuperation time in which cancer cells obtain additional genetic mutations, adjust to a new environment, become resistant to cancer therapies, avoid immune elimination and finally initiate metastasis [2, 3]. Although the mechanisms of cancer dormancy are obscure, increasing evidence indicates that cancer dormancy is context-dependent and the tumor microenvironment (TME) plays an important role in determining the final fate of dormant cancer cells [4–6].

In 1889, Paget proposed that tumor cells and the supportive microenvironment resemble “seed and soil”, both are prerequisites for the development of tumors [7]. Niche-dependence is one of the most important hallmarks of cancer cell dormancy [8]. For example, disseminated tumor cells (DTCs) often colonize and become dormant in some certain locations such as endosteal niche and perivascular niche [4, 5]. Furthermore, the exit of cancer dormancy is also largely dependent on TME. The alteration of the extracellular matrix (ECM) composition can impact the integrin-dependent adhesion signaling which governs the switch from tumor dormancy to metastatic outgrowth [9]. Intratumoral hypoxia can up-regulate uPAR expression and trigger the extracellular signaling, then promote tumor cells epithelial-to-mesenchymal transition (EMT), migration, invasion, and escape from dormancy [10]. Likewise, cellular constituent of TME has been found to participate in this process. Fibroblasts or endothelial tip cells could secrete periostin thus plunging their dormant neighbor DTCs into proliferation [11]. At the primary site, the costimulatory and coinhibitory signal molecules on the surface of the immune cells were major factors in activating dormant tumor cells and leading to lethal recurrence [12].

**Fig. 1:**
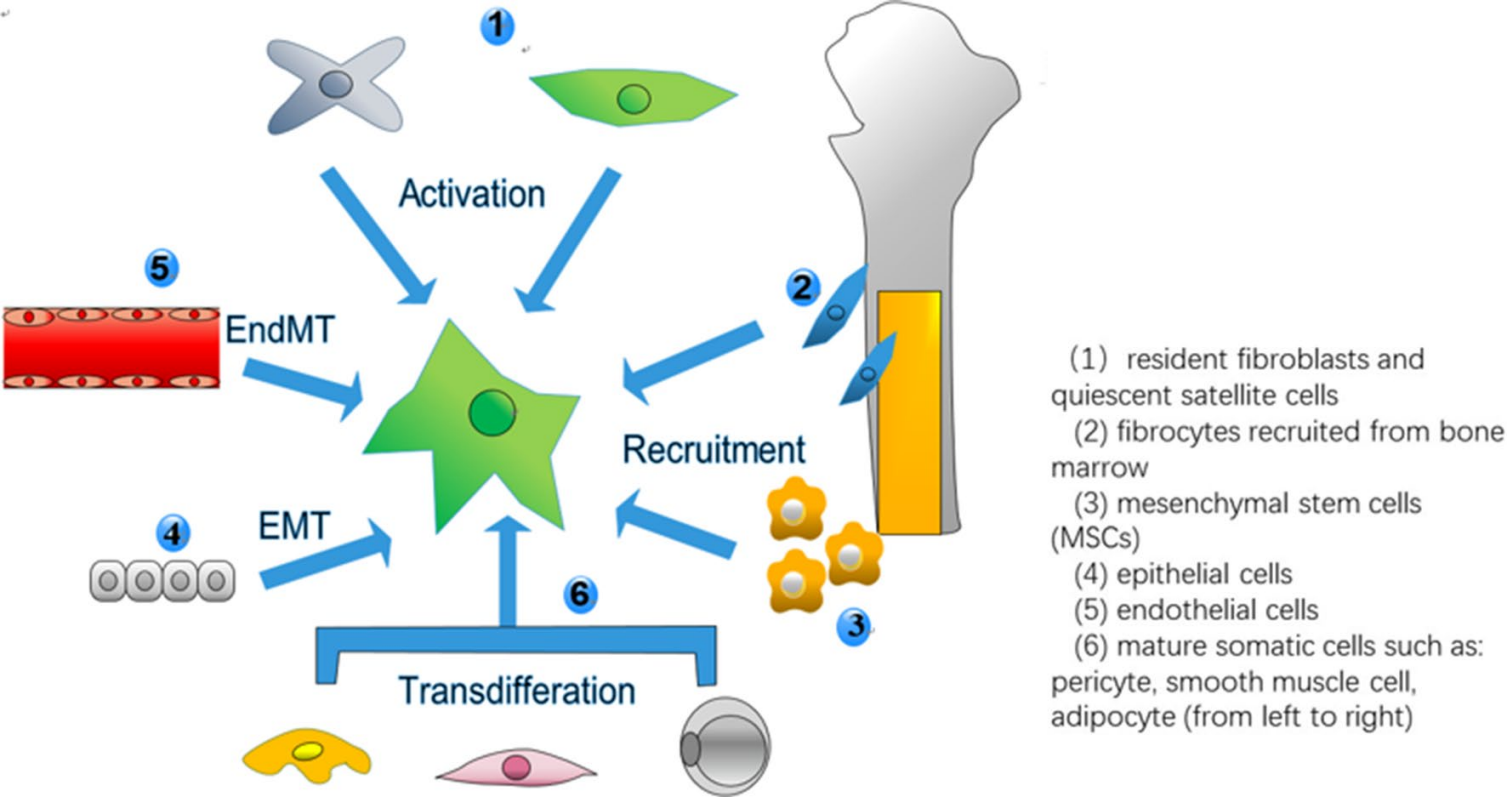
The origins of CAFs. The origins of CAFs are primarily categorized into 6 groups. (1) resident normal fibroblasts or quiescent stellate cells (2) fibrocytes recruited from bone marrow (3) mesenchymal stem cells (4) epithelial cells (5) endothelial cells (6) mature somatic cells such as: pericyte, smooth muscle cell, adipocyte (from left to right)

**Fig. 2:**
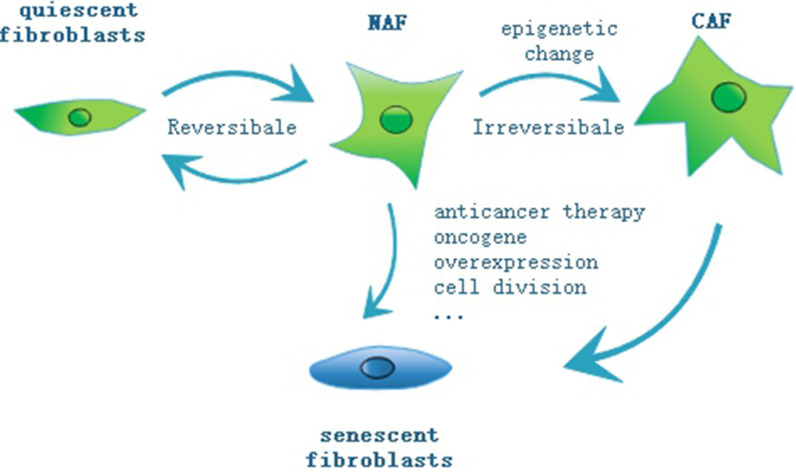
Types and transformations of fibroblasts. This review emphasizes four major types of fibroblasts: quiescent fibroblasts, normal activated fibroblasts (NAFs), cancer associated fibroblasts (CAFs) and senescent fibroblasts. When quiescent fibroblasts are exposed to some stimuli, fibroblasts can become activated reversibly or irreversibly. The reversible one usually appears in the process of wound healing in order to facilitate repair and regeneration. The irreversible one, which is triggered by some perpetual and unabated injurious stimuli, is characterized by epigenetic regulation. Both normal fibroblasts and CAFs can be converted into senescent fibroblasts because of anti-tumor therapies, genetic mutations, natural aging and so forth

As a predominant cell type in TME, fibroblasts participate in multiple biological and pathological processes, such as inflammation, wound healing, metastasis and relapse of tumors [9, 11]. However, the investigation about fibroblasts’ functions in cancer dormancy is little and often contradictory (Table 1). This might be due to their high heterogeneity. Recent studies have proposed numerous potential sources of CAFs and mainly categorized them into 6 groups. The diagram of the origins of CAFs is shown in Fig. 1. Regardless of their origins, their roles in dormancy vary dramatically due to many other factors such as the stage of tumor progression, the tissue context and different cellular stressors [14]. Therefore, CAFs may exhibit some tumor-restraining abilities while their tumor-promoting functions have been widely explored [15]. Normal fibroblasts have been proven to suppress their neighboring aberrant hyperplasia and trap it into a dormant state [16]. But they can also be seduced to become tumor-promoting CAFs [17]. Both normal fibroblasts and CAFs can be converted into senescent fibroblasts because of anti-tumor therapies, genetic mutations, natural aging and so forth [13] (Fig. 2). Senescent fibroblasts commonly function via SASP factors which can both spread senescence and awaken dormant cells in their surroundings [14].

**Table 1:** Functions of fibroblasts based on site of tumors

Site of tumors	Types of fibroblasts	Functions of fibroblasts	
Prostate	Normal lung fibroblasts	Anti-tumor	[96]
	Normal associated fibroblasts	Anti-tumor	[25]
	CAFs	Tumor-promoting	[25, 41]
	Fibroblasts from benign hyperplasia	Anti-tumor	[26]
Skin	Normal dermal fibroblasts	Anti-tumor	[16]
	Senescent fibroblasts	Tumor-promoting	[82, 83]
Pancreas	CAFs	Anti-tumor	[15, 31]
Stomach	CAFs	Tumor-promoting	[46]
Breast	CAFs	Pro-inflammation	[48]
		Chemoresistance	[61]
Ovary	CAFs	Tumor-promoting	[48]
Lung	CAFs	Tumor-promoting	[51]
Colon	CAFs	Chemoresistance	[54]
Liver	CAFs	Pro-angiogenesis	[57]

This review will elucidate the heterogeneity and functional diversity of these three types of fibroblasts in cancer dormancy, providing some new insights into tumor dormancy therapy by targeting fibroblasts.

## Main text

### Normal fibroblasts and dormancy

In normal tissue, quiescent fibroblasts are detected as spindle-like or fusiform shape single cells with front and back extensions in the interstitial space embedded in physiology ECM [18]. They are generally considered to be in a hibernating, quiescent or resting state with negligible metabolic and transcriptomic activity [19]. When they are exposed to some stimuli such as wound healing, persisting inflammation and cancer lesion, fibroblasts can become activated reversibly or irreversibly [20] (Fig. 2). Once activated, fibroblasts gain the ability of proliferation, migration and production of growth factors and ECM as well as some morphological changes [21]. The reversible one usually appears in the process of wound healing in order to facilitate repair and regeneration. When repair is complete, these wound healing-associated fibroblasts can revert into a quiescent state by reprogramming or apoptosis [22].

**Fig. 3:**
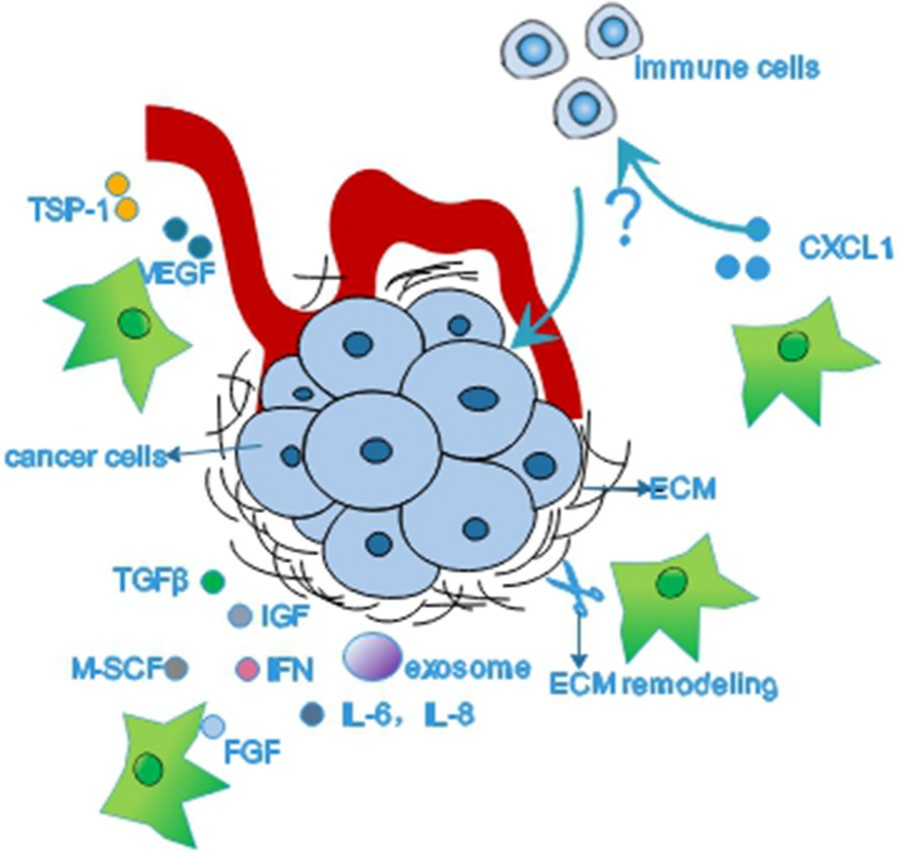
Neighbor suppression phenomena. In the early stage of tumor initiation, the normal fibroblasts impede the growth of neighboring abnormal or transformed cells via cell–cell contact and secreting some soluble factors, which refers to as “neighbor suppression phenomenon”

While in the early stage of tumor initiation, the reversible activated fibroblasts impede the growth of neighboring abnormal or transformed cells via cell–cell contact, which refers to as “neighbor suppression phenomenon” [23] (Fig. 3). Normal lung fibroblasts can inhibit the growth of human prostatic carcinoma cells without contact [24]. Cytokine array revealed that normal activated fibroblasts secreted higher level of TNF-α compared with CAFs, which can counteract the pro-tumor effects of CAFs [25]. Degeorges et al. found out that stromal cells from benign prostatic hyperplasia, composed of fibroblasts and myofibroblasts, produced a large amount of IL-1β, IL-6 and IL-8. And IL-6 was responsible for inhibiting cell growth [26]. Fibroblasts in mouse skin have been shown to inhibit the growth of melanoma cells by reducing p16 and cyclin D1 levels in vivo [16]. These indicate that the normal fibroblast can hinder the cancer cells from unscrupulous development by neighbor suppression-dependent tumor dormancy [27].

The irreversible one, which is triggered by some perpetual and unabated injurious stimuli, is characterized by epigenetic regulation. In this condition, these activated fibroblasts are not only limited to regress but also gain further secretory phenotypes, specialized ECM remodeling ability, robust autocrine activation and dynamic immunomodulatory signaling functions [28]. Fibroblasts irreversibly activated by cancer lesion are termed as CAFs, which drive cancer initiation and activate tumor dormancy [17]. However, the underlying molecular mechanisms of transformation from normal fibroblasts to CAFs still remain unclear.

### CAFs and cancer dormancy

**Fig. 4:**
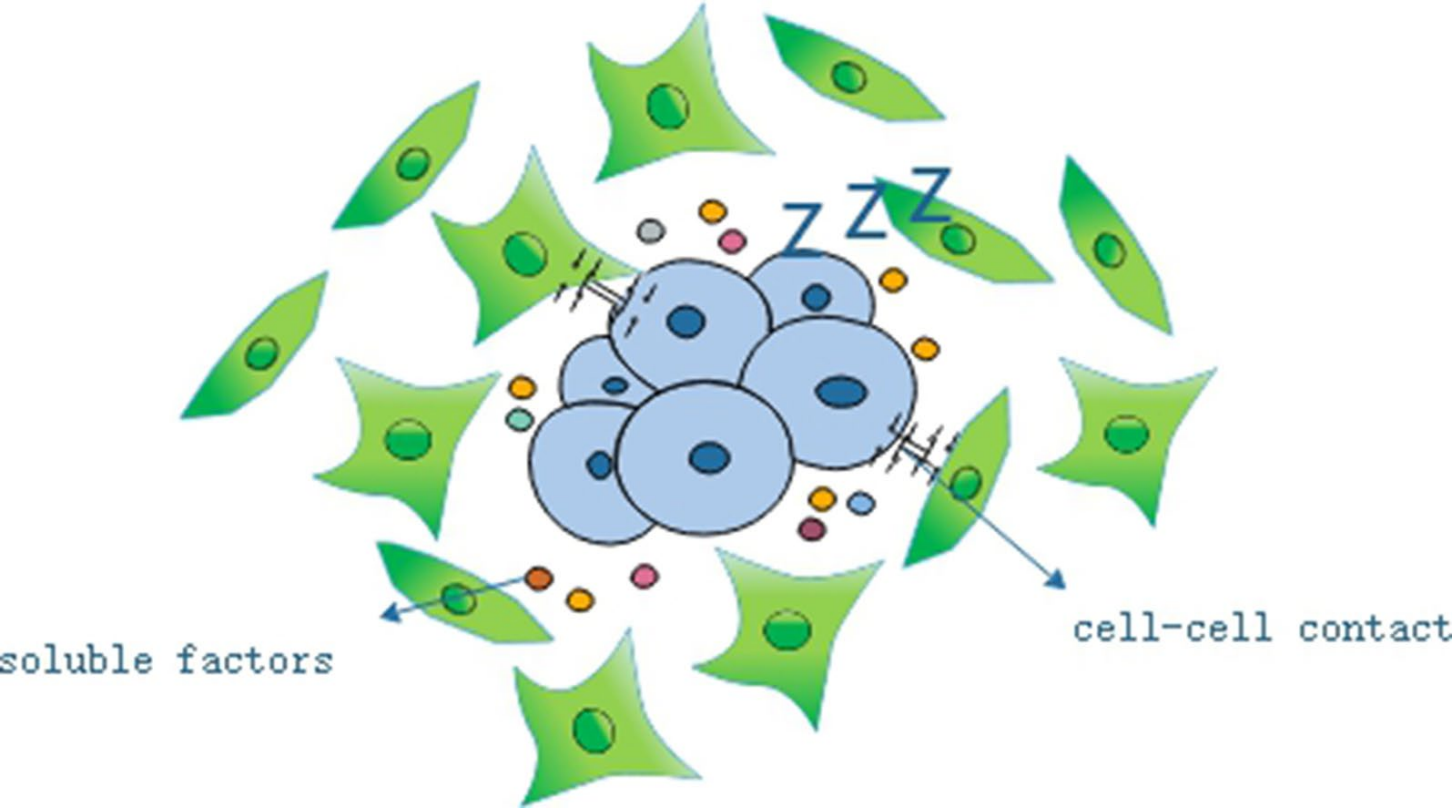
CAFs and cancer dormancy. There is little evidence showing a direct correlation between CAFs and cancer dormancy. We presume that CAFs might influence the state of cancer cells through remodeling ECM or producing exosomes and an array of factors such as TGFβ, IFN, IGF, FGF, M-SCF and IL. CAFs could produce TSP-1 and VEGF to mediate the balance of angiogenesis. It is also possible that CAFs could recruit immune cells then impinge on cancer dormancy

CAFs, which account for the majority of tumor stroma, have been well proven to play a pivotal role in orchestrating cancer progression [29, 30]. They are highly heterogeneous cell populations. Even in the same type of cancer, their gene signatures and protein expression patterns might be distinct, which lead to different or even opposite functions in cancer progression [28]. CAFs commonly exhibit pro-tumorigenic abilities but the depletion of CAFs can cause a more aggressive phenotype in pancreas cancers [15, 25, 31]. It is plausible to assume that there exists a subpopulation of CAFs implicated with the entry or exit of cancer dormancy. However, defining a functional subpopulation of CAFs by cell surface markers still remains challenging although there are a wide range of markers can be applied [32]. The common markers of CAFs have been shown in Table 2. There is little evidence to show the direct correlation between CAFs and cancer dormancy, but some clues could be found to deduce that CAFs might contribute to cancer dormancy or awaken dormant cancer cells from dormancy under different conditions (Fig. 4).

**Table 2:** List of commonly-used CAF markers and their expression

Marker	Some other cells that express the marker	Expression change in CAF
α-SMA	Normal fibroblast, pericyte, smooth muscle cell, cardiomyocyte, breast myoepithelial cell	Upregulated
S100A4 (FSP1)	Normal fibroblast, endothelial cell, neuron, macrophage	Upregulated
Vimentin	Endothelial cell, neuron, adipocyte, breast myoepithelial cell, thyroid gland cell, hematopoietic cell, lymphoid cell	Upregulated
FAP	Activated fibroblast, CD45 + immune cell, smooth muscle cell, epithelial cell, glandular cell in multiple cell	Upregulated
PDGFRα	Astrocyte	Upregulated
PDGFRβ	Normal fibroblast, pericyte, vascular smooth muscle cell, neuron, myocardium	Upregulated
Caveolin (CAV1)	Normal fibroblast, squamous epithelial cell, type I pneumocyte, macrophage, adipocyte,	Upregulated or downregulated
Tenascin C (TNC)	Cancer cell, normal fibroblast	Upregulated
Desmin	Skin fibroblast, pericyte, muscle cell	Downregulated
Cavin-1	Adipocyte, endothelial cell, breast myoepithelial cell, endothelial cell, macrophage, smooth muscle cell	Downregulated
Podoplanin (PDPN)	Lymphatic vessel	Upregulated
Zinc finger E-box binding homeobox-1 (ZEB1)	Adipocyte, smooth muscle cell, endothelial cell, glial cell, myocyte	Upregulated

#### TGF-β

CAFs are the main source of TGF-β, which has been indicated to maintain the dormant state of DTCs [33]. High expression of TGF-β in bone marrow has been shown to contribute to the maintenance of cancer cells’ quiescence in head and neck squamous cell carcinoma (HNSCC) [34]. Yomoto et al. discovered that specific blockade of TGF-β signaling limited the ability of the osteoblasts to induce dormancy of prostate cancer cells through both Gas6 and Axl in bone marrow [35]. Coco, a secreted antagonist of BMP (a family member of TGF-β) ligands, was proven to reactivate dormant breast cancer cells in the lung, which did not occur in the bone or brain, suggesting an organ-specific modulation of cancer cell dormancy [36]. However, TGF-β can also facilitate the proliferation of cancer cells. Qin et al. found out that TGF-β3 enabled to promote the growth, migration, and invasion of HNSCC through inducing the expression of matrix-specific protein periostin [37]. In advanced-stage cancers of cervix, TGF-β1 could favor tumor growth. While it was shown to restrain tumor progression in precancerous lesions at early stage [38]. This difference indicates that TGF-β may induce dormancy to limit tumor growth at the initial stage, and then under some conditions, TGF-β may activate the dormant cancer cells to metastasize. Further study is required to explore that under what condition TGF-β could serve as a cancer dormancy activator or an inducer.

#### Interferon (IFN)

IFN-β, which impinges on immunological dormancy of cancers, is primarily secreted by fibroblasts. Lan et al. published that persistent activation of the IFN-β/IFNAR/IRF7 signaling axis was crucial to the chemotherapy-induced dormancy in ER^−^ breast cancer cells [39]. Liu et al. found out that IFN-β could instigate tumor-repopulating cells (TRC) of melanoma into dormancy through an indolamine 2, 3-dioxygenase/kynurenine/aryl hydrocarbon receptor/p27–dependent (IDO/Kyn/AhR/p27-dependent) pathway [40].

IFN-γ is commonly from T cells but there was a study demonstrated that CAFs could produce IFN-γ, which promotes expression of stem cell markers in prostate cancer epithelial cells [41]. IFN-γ enables to induce cancer cell dormancy through the activation of STAT1. Kortylewski et al. published that the activation of the IFN-γ/STAT1 pathway could drive melanoma cell into dormancy via downregulation of cyclin E and cyclin A [42]. Another research reported that the IFN-γ-induced activation of STAT1 could cause G1-arrest of melanoma cells through upregulating microRNA-29 family which target at CDK6 [43]. In fibrosarcoma, STAT1 activated by IFN-γ could directly interact with cyclins D1, D2, D3 and CDK4, leading to cell cycle arrest [44]. IFN-γ can also mediate cancer cell dormancy in a STAT1-independent way and the effects of IFN-γ might be partly dependent on the properties of the cancer cells. Liu et al. proposed that IFN-γ commonly caused apoptosis in differentiated tumor cells while induced TRCs to enter dormancy through IDO/Kyn/AhR/p27-dependent pathway [45].

#### Interleukin (IL)

Zhai et al. revealed that CAFs from human gastric cancer could secrete more IL-8 compared with normal fibroblasts, then conferring cancer cell chemoresistance [46]. Of note, IL-8 was shown to promote the proliferation of dormant breast cancer cells in liver through IL-8Rb/CXCR2 [47]. In human breast and ovarian tumors, it has been shown that a high level of IL-6 produced by CAFs was a vital part to facilitate the stroma inflammation [48]. In another research, IL-6 treatment could help breast cancer cells exit from dormancy mediated by hormone therapy by activation of Notch3 [49].

#### Chemokines

CAFs were the main source of chemokines such as CXCL5 [50] and CXCL1 [51]. The addition of exogenous recombinant CXCL5 protein could promote the proliferation of quiescent breast cancer cells under healthy bone co-cultures via binding to CXCR2 [52]. It is generally accepted that chemokines are significant in the recruitment of immune cells which also play a crucial role in cancer dormancy, but the research about the direct effect of CAFs on immune cells is little. Kumar et al. demonstrated that an increasing expression of CXCL1 from CAFs could recruit polymorphonuclear myeloid-derived suppressor cells (PMN-MDSC) then neutralized the anti-tumor effect of CSF1 receptor blockage [51]. This might leave us a hint that CAFs could mediate cancer dormancy with the help of immune cells.

#### Other cytokines

CAFs can produce a wide range of growth factors such as fibroblast growth factor (FGF), epidermal growth factor (EGF) and vascular endothelial growth factor (VEGF). Barrios et al. found out that FGF-2 could induce dormancy of breast cancer cells by activating PI3K/Akt pathway in an integrin α5β1-dependent or independent way [53]. In colorectal cancer, the administration of cetuximab could induce EGF secretion of CAFs only, exclusive of cancer cells and normal fibroblasts [54]. And Clark et al. established an ex vivo hepatic microphysiological system in which they used EGF and lipopolysaccharide to activate the residual dormant breast cancer cells after chemotherapy [55]. The angiogenetic dormancy is maintained by the equilibrium of anti- and pro-angiogenetic factors such as VEGF and thrombospondin-1 (TSP-1), respectively [56]. Huang et al. discovered that the secretion of VEGF form CAFs promoted angiogenesis of human umbilical vein endothelial cells via EZH2/VASH1 pathway in hepatocellular carcinoma [57]. In invasive breast ductal carcinomas, TSP-1 is primarily produced by fibroblasts presenting in desmoplastic areas instead of cancer cells themselves [58]. This might suggest that CAFs are crucial to orchestrate the balance of anti- and pro-angiogenic factors and further get involved in angiogenetic dormancy.

However, CAFs might influence cancer dormancy by producing an array of substances rather than a single factor. Mao eta al. uncovered that the elevated expressions of IL-8, VEGF and IGF in the microenvironment could rescue the DIRAS3-induced autophagic cell death and lead to dormancy in ovarian cancer xenografts model [59]. The upregulation of ARHI enables to induce autophagic cell death in vitro but dormancy in xenografts in ovarian cancer because the growth factors (IGF-1, M-CSF), angiogenic factors (VEGF, IL-8), and matrix proteins from microenvironment could reduce the effect of ARHI [60]. In human breast and ovarian tumors, it has been shown that CAFs could facilitate the stroma inflammation by expressing high levels of IL-6, COX-2 and CXCL1 [48]. Apart from soluble factors, Sansone et al. [61] revealed that CAFs could horizontally transfer extracellular vesicles, which contain the full mitochondrial genome, to hormonal therapy-resistant breast cancer cells and facilitate an exit from metabolic quiescence in both vitro and vivo (Fig. 4).

#### ECM

CAFs have high capacity of ECM synthesis and remodeling during desmoplastic reaction. Stiffness of tumor stroma, which is mainly regulated by CAFs, is associated with cancer cell dormancy. For instance, Lv et al. established a 3D fibrin gel system and discovered that increased matrix stiffness could induce TRCs of melanoma into dormancy by mediating the translocation of cytosolic Cdc42 into nucleus [62]. Cultured in an ECM-mimicking semi-solid Matrigel, A549 lung cancer cells exhibited a significant decrease in the expression of uPA, uPAR, MMP2, MMP7, MMP9 and CXCR4 and entered into a drug-resistant dormancy state. And the inhibition of the ERK1/2 and PI3K/Akt pathways might get involved in this process [63]. The composition of ECM could also determine the fate of the dormant cancer cells. Barkan et al. showed that both type I collagen and fibronectin could drive dormant D2.0R cells into proliferation through β1-integrin induced PI3K/Akt and FAK activation [64, 65]. Tenascin-C, which is increasingly expressed by activated fibroblasts in the metastatic niche, could inhibit pro-dormancy cellular reprogramming thus promoting proliferative signaling through sequestering TGFβ in an inactive state [66, 67].

### Senescent fibroblasts and cancer dormancy

#### Dormancy versus senescence

Cellular senescence is a stress-response cell cycle program mostly occurred in malignant cells after numerous anti-cancer therapies [68]. Most senescent cells are metabolically active and able to secrete various inflammatory factors, extracellular-modifying substances and growth factors, collectively termed as the senescence-associated phenotype (SASP), that influence tumor progression [14]. Apart from the special makers such as senescence-associated β-galactosidase and signaling molecules like Bmi-1, p16, p21, a prim difference is that senescence used to be defined as an unrecoverable state in which the further expansion of cancer cells is permanently terminated while cancer dormancy is reversible [2]. However, increasing studies began to challenge this perspective and support that senescence may not be recognized as a completely distinct process from dormancy. Recent evidence indicates that therapy-induced senescence is a reversible condition as well [68]. It also shares some molecular signals with dormancy (for example, p38/MAPK, mTOR signaling). Additionally, autophagy, which is considered as a prerequisite for senescence, is important in cancer dormancy [69]. Tareq et al. [70] speculated that senescence may be one form of cancer dormancy, whereby cancer cells circumvent the direct cytotoxic influence of therapy, maintain the self-renewal ability and eventually contribute to disease relapse. Of note, dormancy is demonstrated to be not a uniform state but with graded depth in normal tissue, more specifically, shallow dormant cells manifest a higher tendency to go back into the cell cycle than deep dormant cells [71]. In addition, lysosome, which plays a critical role in senescence, is found to function as a dimmer switch to mediate the quiescence depth in rat embryonic fibroblasts [72, 73]. Dose cancer dormancy exhibit graded depth as well? Can senescence be considered as a deeper-depth dormancy? Is it possible that lysosome takes participate in the switch between dormancy and senescence? The answers of above questions are still up in the air.

#### Senescent fibroblasts and cancer dormancy

Stroma senescence in cancers could be induced by many factors such as sequential rounds of cellular division (replicative senescence), anti-cancer therapies (therapy-induced senescence) and overexpression of oncogene (oncogene-induced senescence) [14]. Tivari et al. demonstrated that bone marrow stroma secretory senescence, which was induced by oxidation, hypoxia and estrogen deprivation with hydrogen peroxide (H_2_O_2_), carbonyl-cyanide m-chlorophenylhydrazzone (CCCP) and Fulvestrant, could awake the dormant estrogen receptor positive (ER +) breast cancer cells by secreting some inflammatory cytokines such as IL-6, IL-8, TNF α and TGFβ1 [74]. This shows that stroma senescence has association with cancer dormancy. Senescent fibroblasts, no matter derived from normal fibroblasts or CAFs, enter into a permanent cell cycle arrest but still have significant but paradoxical effects on cancer initiation and progression primarily through SASP [14, 75]. IOS fibroblasts can secrete TGF-β and IL-6 to induce neighboring cells into senescence, while in the meantime producing IL-1α, which in turn triggers SASP in a paracrine manner [76, 77]. Beyond that, several animal models have demonstrated that either senescent stroma in early stages of tumor progression or tumor cells are able to express SASP factors, which can suppress tumor growth with the help of immune response [78, 79]. Further, irradiated fibroblasts could rescue the decreased secretion of IL-23 of dendritic cells [80]. And elevated expression of IL-23 could lead to cancer cell dormancy in esophageal squamous carcinoma through the Wnt/Notch pathway [81]. Although senescent stroma could exhibit tumor-suppressive ability, its tumor-promoting effects are more common. In a stromal specific senescence model, Ruhland et al. discovered that senescent fibroblasts in the skin could inhibit CD8 + T cell activity via recruiting suppressive immature granulocytes no matter whether tumor cells existed [82]. Both in vitro and in vivo, it has been demonstrated that senescent fibroblasts induced by persisting CDK4/6 inhibition can efficiently improve melanoma cell growth [83]. Importantly, Faget et al. proposed a hypothesis that these tumor-promoting SASP factors secreted by senescent fibroblasts could drive the dormant neighboring cancer cell to awaken [14]. This suggests that senescent fibroblasts could play a tumor-promoting role by activating dormancy or a tumor-suppressive role through inducing dormancy.

### Circulation CAFs and activation of cancer dormancy

**Fig. 5:**
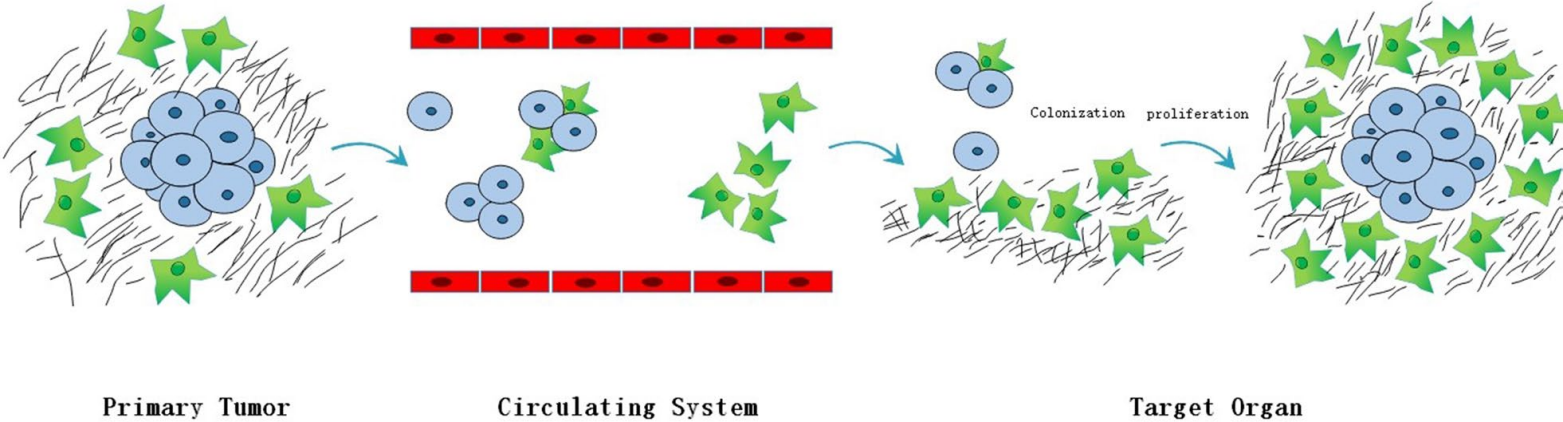
Circulation CAFs. CAFs from primary tumor can intravasate into blood vessels even before CTCs appearing and are transported to target organ to modify the metastatic microenvironment into a hospitable location for colonization of tumor cells. CAFs can also be a part of CTC clusters and enhance the metastatic ability of tumor cells. Once arrived at metastasis loci, c-CAFs might prompt tumor cells to escape dormancy and initiate early growth

Increasing researchers presume that c-CAFs can also play a crucial role in tumor dormancy. Duda et al. [84] reported that metastatic cancer cells could carry some “soil” from their initial microenvironment. CAFs from the primary tumor can be transported to lung to modify the metastatic microenvironment into a hospitable location for colonization of tumor cells. The first function of c-CAFs is to protect tumor cells from apoptosis. Once arrived at metastasis loci, c-CAFs prompt tumor cells to escape dormancy and initiate early growth. Zhou et al*.* [85] proposed that SMC1A, a mucin subunit, could influence the microenvironment remodeling of CAFs and boost the tumor initiation. Colorectal cancer cells, which express a high level of SMC1A, can recruit c-CAFs to improve their ability of migration and survival, thereby developing metastasis. In a mouse model of breast cancer, CTC cluster exhibits a more enhanced metastatic ability than solitary or single CTCs [86]. Except for CTCs, peripheral blood also contains circulation CAFs derived from TME [87]. In human xenotransplant model of breast cancer, along with tumor development and metastasis, the amounts of CTCs, CTCs cluster and c-CAF/CTC co-cluster in peripheral blood increase. In peripheral blood, CAFs, which appear antedate CTCs, exist in the form of solitary CAFs, a part of CTC cluster and CAFs cluster [88]. The mouse metastatic model of lung cancer has testified that c-CAFs can extravasate from circulatory system with or without tumor cells then colonize in pre-metastatic and metastatic microenvironment, which facilitates immune evasion, metastatic colonization and tumor growth [84]. Ao et al*.* [89] discovered that c-CAFs could be detected in stage IV breast cancer patients with metastasis but not in stage I patients without indication for relapse, while both of them could detect the existence of CTCs (Fig. 5).

### Targeting fibroblasts and cancer dormancy

There are mainly three strategies to target cancer cell dormancy: sleeping strategy, awakening strategy and killing strategy [1]. Literally, sleeping strategy aims at maintaining the dormancy of cancer cells. Awakening strategy is to activate dormant cells, thereby increasing their sensitivity towards chemotherapy. Killing strategy is intended for eliminating dormant cancer cells. In order to maintain dormancy, we could remove or normalize tumor-promoting fibroblasts and keep the tumor-restraining ones. It is also plausible to imitate how the fibroblasts in the dormant niches function thus constraining cancer cells into a dormant state. As to awakening strategy, pro-tumorigenic fibroblasts could be applied to force dormant cancer cells to re-enter the cell cycle.

Chemotherapy resistance is the biggest obstacle of killing dormant cancer cells, and fibroblasts can be considered as co-conspirators. For example, the drug-scavenging ability of CAFs can entrap the anti-tumor drug inside and reduce its potency [90]. The anti-tumor effects of CSF receptor blockade can be neutralized by CAFs through immune recruitment of PMN-MDSCs [51]. Therefore, targeting at CAFs might be an efficient approach to increase the efficacy of anti-tumor drugs and ultimately overcome chemoresistance [91]. It is true that some drugs targeting CAFs-derived molecular signals, such as Plerixafor and Galunisertib, have shown to be effective in clinical trials, but it is also true that they are commonly spatial–temporal and case-dependent [92]. Moreover, some other preclinical researches also demonstrated that the indiscriminate targeting or elimination of stromal fibroblasts might lead to a more aggressive phenotype [28, 93]. Recently, some investigations began to discuss the modulation/silencing of fibroblasts rather than physical elimination. In esophageal adenocarcinoma, phosphodiesterase type 5 inhibitor (PDE5i) has been proven to enhance the efficacy of chemotherapy by attenuating the myofibroblast phenotype of fibroblasts. Meanwhile, the safety and effect of PDE5i were confirmed in a patient-derived xenograft model [94]. Anbil et al*.* discovered that the combination therapies of oxaliplatin and photodynamic therapy with metformin could negate CAF-induced treatment resistant through partially modulating the redox metabolism based on an adherent 3D culture model of pancreatic cancer [95].

## Conclusions

Fibroblast plays a complex and multifaceted character in cancer dormancy. Different fibroblasts populations might play conflicting roles in cancer dormancy, it is unreasonable to simply define them as foes or friends. With the development of tumors, fibroblasts show their versatile functions and dynamically evolve with the cancer cells, whereas the mechanisms that can switch the “flavor” of fibroblasts still remain obscure [75].

Cancer dormancy tend to shift into lethal recurrence, posing severe challenges to clinical treatment. However, there still lacks effective approach to overcome this problem. Current paradigms of cancer-centric therapeutics are usually not sufficient to eradicate the malignancy, and therapies targeting at stroma attract more and more attention. Fibroblasts, which comprise the majority of tumor stroma, might be a new breach. However, there are many problems hindering the application of fibroblast-targeted therapy. For instance, which CAFs subpopulations play the most important role in tumor biology and how to identify them? As CAFs can secrete most factors of SASP, could senescent fibroblasts in cancers be considered as a special subpopulation of CAFs? How to protect other normal cells from fibroblast-target therapy? What are the underlying mechanisms that govern the biological behavior of CAFs and how to pass it into the next generation stably?

Although the biology and function of fibroblasts are elusive, as the major cell in TME, fibroblasts might be the Achilles heel of cancer dormancy and pave a new way for cancer therapy.

## Data Availability

All available.

## Abbreviations

CAFs: Cancer associated fibroblasts
TME: Tumor microenvironment
DTCs: Disseminated tumor cells
ECM: Extracellular matrix
TRC: Tumor-repopulating cells
PMN-MDSC: Polymorphonuclear myeloid-derived suppressor cells
FGF: Fibroblast growth factor
EGF: Epidermal growth factor
VEGF: Vascular endothelial growth factor
TSP-1: Thrombospondin-1
EMT: Epithelial-to-mesenchymal transition
SASP: Senescence-associated phenotype
PDE5i: Phosphodiesterase type 5 inhibitor

## References

[1] Recasens A, Munoz L (2019). Targeting cancer cell dormancy. Trends Pharmacol Sci.

[2] Aguirre-Ghiso JA (2007). Models, mechanisms and clinical evidence for cancer dormancy. Nat Rev Cancer.

[3] Bartosh TJ, Ullah M, Zeitouni S, Beaver J, Prockop DJ (2016). Cancer cells enter dormancy after cannibalizing mesenchymal stem/stromal cells (MSCs). Proc Natl Acad Sci.

[4] Carpenter RA, Kwak J-G, Peyton SR, Lee J (2018). Implantable pre-metastatic niches for the study of the microenvironmental regulation of disseminated human tumour cells. Nat Biomed Eng.

[5] Goddard ET, Bozic I, Riddell SR, Ghajar CM (2018). Dormant tumour cells, their niches and the influence of immunity. Nat Cell Biol.

[6] Manjili MH (2017). Tumor dormancy and relapse: from a natural byproduct of evolution to a disease state. Can Res.

[7] Paget S (1889). The distribution of secondary growths in cancer of the breast. Lancet.

[8] Phan TG, Croucher PI (2020). The dormant cancer cell life cycle. Nat Rev Cancer.

[9] Kai F, Drain AP, Weaver VM (2019). The extracellular matrix modulates the metastatic journey. Dev Cell.

[10] Sullivan R, Graham CH. Hypoxia-driven selection of the metastatic phenotype (0167–7659 (Print)). 10.1007/s10555-007-9062-2 17458507

[11] Malanchi I, Santamariamartinez A, Susanto E, Peng H, Lehr H, Delaloye J (2012). Interactions between cancer stem cells and their niche govern metastatic colonization. Nature.

[12] Teng MW, Swann JB, Koebel CM, Schreiber RD, Smyth MJ (2008). Immune-mediated dormancy: an equilibrium with cancer. J Leukoc Biol.

[13] Krenning G, Zeisberg EM, Kalluri R (2010). The origin of fibroblasts and mechanism of cardiac fibrosis. J Cell Physiol.

[14] Faget DV, Ren Q, Stewart SA (2019). Unmasking senescence: context-dependent effects of SASP in cancer. Nat Rev Cancer.

[15] Özdemir BC, Pentcheva-Hoang T, Carstens JL, Zheng X, Wu C-C, Simpson TR (2014). Depletion of carcinoma-associated fibroblasts and fibrosis induces immunosuppression and accelerates pancreas cancer with reduced survival. Cancer Cell.

[16] Zhou L, Yang K, Randall Wickett R, Zhang Y (2016). Dermal fibroblasts induce cell cycle arrest and block epithelial–mesenchymal transition to inhibit the early stage melanoma development. Cancer Med.

[17] Dasari S, Fang Y, Mitra AK (2018). Cancer associated fibroblasts: naughty neighbors that drive ovarian cancer progression. Cancers.

[18] Tarin D, Croft CB (1969). Ultrastructural features of wound healing in mouse skin. J Anat.

[19] Kalluri R (2016). The biology and function of fibroblasts in cancer. Nat Rev Cancer.

[20] Yeo S-Y, Lee K-W, Shin D, An S, Cho K-H, Kim S-H (2018). A positive feedback loop bi-stably activates fibroblasts. Nat Commun.

[21] Wang XM, Yu DMT, McCaughan GW, Gorrell MD (2005). Fibroblast activation protein increases apoptosis, cell adhesion, and migration by the LX-2 human stellate cell line. Hepatology.

[22] Yin C, Evason KJ, Asahina K, Stainier DY (2013). Hepatic stellate cells in liver development, regeneration, and cancer. J Clin Investig.

[23] Stoker MG, Shearer M, O'Neill C (1966). Growth inhibition of polyoma-transformed cells by contact with static normal fibroblasts. J Cell Sci.

[24] Kirk D, Szalay MF, Kaighn ME (1981). Modulation of growth of a human prostatic cancer cell line (PC-3) in agar culture by normal human lung fibroblasts. Cancer Res.

[25] Paland N, Kamer I, Kogan-Sakin I, Madar S, Goldfinger N, Rotter V (2009). Differential influence of normal and cancer-associated fibroblasts on the growth of human epithelial cells in an in vitro cocultivation model of prostate cancer. Mol Cancer Res.

[26] Degeorges A, Tatoud R, Fauvel-Lafeve F, Podgorniak MP, Millot G, de Cremoux P (1996). Stromal cells from human benign prostate hyperplasia produce a growth-inhibitory factor for LNCaP prostate cancer cells, identified as interleukin-6. Int J Cancer.

[27] Folkman J, Kalluri R (2004). Cancer without disease. Nature.

[28] Alkasalias T, Moyano-Galceran L, Arsenian-Henriksson M, Lehti K (2018). Fibroblasts in the tumor microenvironment: shield or spear?. Int J Mol Sci.

[29] Marsh T, Pietras K, McAllister SS (2013). Fibroblasts as architects of cancer pathogenesis. Biochim Biophys Acta (BBA) Mol Basis Dis.

[30] Östman A, Augsten M (2009). Cancer-associated fibroblasts and tumor growth—bystanders turning into key players. Curr Opin Genet Dev.

[31] Rhim AD, Oberstein PE, Thomas DH, Mirek ET, Palermo CF, Sastra SA (2014). Stromal elements act to restrain, rather than support, pancreatic ductal adenocarcinoma. Cancer Cell.

[32] Chen X, Song E (2019). Turning foes to friends: targeting cancer-associated fibroblasts. Nat Rev Drug Discov.

[33] Bhowmick N, Chytil A, Plieth D, Gorska A, Dumont N, Shappell S (2004). TGF-beta signaling in fibroblasts modulates the oncogenic potential of adjacent epithelia. Science.

[34] Bragado P, Estrada Y, Parikh F, Krause S, Capobianco C, Farina HG (2013). TGF-beta2 dictates disseminated tumour cell fate in target organs through TGF-beta-RIII and p38alpha/beta signalling. Nat Cell Biol.

[35] Yumoto K, Eber MR, Wang J, Cackowski FC, Decker AM, Lee E (2016). Axl is required for TGF-β2-induced dormancy of prostate cancer cells in the bone marrow. Sci Rep.

[36] Gao H, Chakraborty G, Lee-Lim AP, Mo Q, Decker M, Vonica A (2012). The BMP inhibitor Coco reactivates breast cancer cells at lung metastatic sites. Cell.

[37] Qin X, Yan M, Zhang J, Wang X, Shen Z, Lv Z, et al. TGFβ3-mediated induction of Periostin facilitates head and neck cancer growth and is associated with metastasis. Sci Rep. 2016;6(1). 10.1038/srep20587 PMC4746667 26857387

[38] Zhu H, Luo H, Shen Z, Hu X, Sun L, Zhu X (2016). Transforming growth factor-β1 in carcinogenesis, progression, and therapy in cervical cancer. Tumor Biol.

[39] Lan Q, Peyvandi S, Duffey N, Huang YT, Barras D, Held W (2019). Type I interferon/IRF7 axis instigates chemotherapy-induced immunological dormancy in breast cancer. Oncogene.

[40] Liu Y, Lv J, Liu J, Liang X, Jin X, Xie J (2018). STAT3/p53 pathway activation disrupts IFN-beta-induced dormancy in tumor-repopulating cells. J Clin Invest.

[41] Liao C-P, Chen L-Y, Luethy A, Kim Y, Kani K, MacLeod AR, et al. Androgen receptor in cancer-associated fibroblasts influences—stemness in cancer cells. Endocr Relat Cancer. 2017. 10.1530/ERC-16-0138 PMC5453797 28264911

[42] Kortylewski M, Komyod W, Kauffmann ME, Bosserhoff A, Heinrich PC, Behrmann I (2004). Interferon-gamma-mediated growth regulation of melanoma cells: involvement of STAT1-dependent and STAT1-independent signals. J Invest Dermatol.

[43] Schmitt MJ, Philippidou D, Reinsbach SE, Margue C, Wienecke-Baldacchino A, Nashan D (2012). Interferon-γ-induced activation of signal transducer and activator of transcription 1 (STAT1) up-regulates the tumor suppressing microRNA-29 family in melanoma cells. Cell Commun Signal.

[44] Dimco G, Knight RA, Latchman DS, Stephanou A (2010). STAT1 interacts directly with cyclin D1/Cdk4 and mediates cell cycle arrest. Cell Cycle.

[45] Liu Y, Liang X, Yin X, Lv J, Tang K, Ma J (2017). Blockade of IDO-kynurenine-AhR metabolic circuitry abrogates IFN-gamma-induced immunologic dormancy of tumor-repopulating cells. Nat Commun.

[46] Zhai J, Shen J, Xie G, Wu J, He M, Gao L (2019). Cancer-associated fibroblasts-derived IL-8 mediates resistance to cisplatin in human gastric cancer. Cancer Lett.

[47] Khazali AS, Clark AM, Wells A (2018). Inflammatory cytokine IL-8/CXCL8 promotes tumour escape from hepatocyte-induced dormancy. Br J Cancer.

[48] Erez N, Glanz S, Raz Y, Avivi C, Barshack I (2013). Cancer associated fibroblasts express pro-inflammatory factors in human breast and ovarian tumors. Biochem Biophys Res Commun.

[49] Sansone P, Ceccarelli C, Berishaj M, Chang Q, Rajasekhar VK, Perna F (2016). Self-renewal of CD133(hi) cells by IL6/Notch3 signalling regulates endocrine resistance in metastatic breast cancer. Nat Commun.

[50] Zhang W, Wang H, Sun M, Deng X, Wu X, Ma Y (2020). CXCL5/CXCR2 axis in tumor microenvironment as potential diagnostic biomarker and therapeutic target. Cancer Commun (Lond).

[51] Kumar V, Donthireddy L, Marvel D, Condamine T, Wang F, Lavilla-Alonso S (2017). Cancer-associated fibroblasts neutralize the anti-tumor effect of CSF1 receptor blockade by inducing PMN-MDSC infiltration of tumors. Cancer Cell.

[52] Romero-Moreno R, Curtis KJ, Coughlin TR, Miranda-Vergara MC, Dutta S, Natarajan A (2019). The CXCL5/CXCR2 axis is sufficient to promote breast cancer colonization during bone metastasis. Nat Commun.

[53] Barrios J, Wieder R (2009). Dual FGF-2 and intergrin alpha5beta1 signaling mediate GRAF-induced RhoA inactivation in a model of breast cancer dormancy. Cancer Microenviron.

[54] Garvey CM, Lau R, Sanchez A, Sun RX, Fong EJ, Doche ME (2020). Anti-EGFR therapy induces EGF secretion by cancer-associated fibroblasts to confer colorectal cancer chemoresistance. Cancers (Basel).

[55] Clark AM, Kumar MP, Wheeler SE, Young CL, Venkataramanan R, Stolz DB (2018). A model of dormant-emergent metastatic breast cancer progression enabling exploration of biomarker signatures. Mol Cell Proteomics.

[56] Natale G, Bocci G (2018). Does metronomic chemotherapy induce tumor angiogenic dormancy? A review of available preclinical and clinical data. Cancer Lett.

[57] Huang B, Huang M, Li Q (2019). Cancer-associated fibroblasts promote angiogenesis of hepatocellular carcinoma by VEGF-mediated EZH2/VASH1 pathway. Technol Cancer Res Treat.

[58] Clezardin P, Frappart L, Clerget M, Pechoux C, Delmas PD (1993). Expression of thrombospondin (TSP1) and its receptors (CD36 and CD51) in normal, hyperplastic, and neoplastic human breast. Cancer Res.

[59] Mao W, Peters HL, Sutton MN, Orozco AF, Pang L, Yang H (2019). The role of vascular endothelial growth factor, interleukin 8, and insulinlike growth factor in sustaining autophagic DIRAS3-induced dormant ovarian cancer xenografts. Cancer.

[60] Lu Z, Luo RZ, Lu Y, Zhang X, Yu Q, Khare S (2008). The tumor suppressor gene ARHI regulates autophagy and tumor dormancy in human ovarian cancer cells. J Clin Invest.

[61] Sansone P, Savini C, Kurelac I, Chang Q, Amato LB, Strillacci A (2017). Packaging and transfer of mitochondrial DNA via exosomes regulate escape from dormancy in hormonal therapy-resistant breast cancer. Proc Natl Acad Sci USA.

[62] Liu Y, Lv J, Liang X, Yin X, Zhang L, Chen D (2018). Fibrin stiffness mediates dormancy of tumor-repopulating cells via a Cdc42-driven Tet2 epigenetic program. Can Res.

[63] Keeratichamroen S, Lirdprapamongkol K, Svasti J (2018). Mechanism of ECM-induced dormancy and chemoresistance in A549 human lung carcinoma cells. Oncol Rep.

[64] Barkan D, Kleinman H, Simmons JL, Asmussen H, Kamaraju AK, Hoenorhoff MJ (2008). Inhibition of metastatic outgrowth from single dormant tumor cells by targeting the cytoskeleton. Cancer Res.

[65] Barkan D, El Touny LH, Michalowski AM, Smith JA, Chu I, Davis AS (2010). Metastatic growth from dormant cells induced by a col-I-enriched fibrotic environment. Cancer Res.

[66] Oskarsson T, Acharyya S, Zhang XH, Vanharanta S, Tavazoie SF, Morris PG (2011). Breast cancer cells produce tenascin C as a metastatic niche component to colonize the lungs. Nat Med.

[67] Massague J (2012). TGFbeta signalling in context. Nat Rev Mol Cell Biol.

[68] Milanovic M, Fan DN, Belenki D, Däbritz JHM, Zhao Z, Yu Y (2018). Senescence-associated reprogramming promotes cancer stemness. Nature.

[69] Young AR, Narita M, Ferreira M, Kirschner K, Sadaie M, Darot JF (2009). Autophagy mediates the mitotic senescence transition. Genes Dev.

[70] Saleh T, Tyutyunyk-Massey L, Gewirtz DA (2019). Tumor cell escape from therapy-induced senescence as a model of disease recurrence after dormancy. Can Res.

[71] Rodgers JT, King KY, Brett JO, Cromie MJ, Charville GW, Maguire KK (2014). mTORC1 controls the adaptive transition of quiescent stem cells from G 0 to G Alert. Nature.

[72] Fujimaki K, Li R, Chen H, Della Croce K, Zhang HH, Xing J (2019). Graded regulation of cellular quiescence depth between proliferation and senescence by a lysosomal dimmer switch. Proc Natl Acad Sci.

[73] Tonnessen-Murray CA, Frey WD, Rao SG, Shahbandi A, Ungerleider NA, Olayiwola JO (2019). Chemotherapy-induced senescent cancer cells engulf other cells to enhance their survival. J Cell Biol.

[74] Tivari S, Lu H, Dasgupta T, De Lorenzo MS, Wieder R (2018). Reawakening of dormant estrogen-dependent human breast cancer cells by bone marrow stroma secretory senescence. Cell Commun Signal.

[75] Goruppi S, Dotto GP (2013). Mesenchymal stroma: primary determinant and therapeutic target for epithelial cancer. Trends Cell Biol.

[76] Acosta JC, Banito A, Wuestefeld T, Georgilis A, Janich P, Morton JP (2013). A complex secretory program orchestrated by the inflammasome controls paracrine senescence. Nat Cell Biol.

[77] Nelson G, Kucheryavenko O, Wordsworth J, von Zglinicki T (2018). The senescent bystander effect is caused by ROS-activated NF-κB signalling. Mech Ageing Dev.

[78] Xue W, Zender L, Miething C, Dickins RA, Hernando E, Krizhanovsky V (2007). Senescence and tumour clearance is triggered by p53 restoration in murine liver carcinomas. Nature.

[79] Iannello A, Thompson TW, Ardolino M, Lowe SW, Raulet DH (2013). p53-dependent chemokine production by senescent tumor cells supports NKG2D-dependent tumor elimination by natural killer cells. J Exp Med.

[80] Malecka A, Wang Q, Shah S, Sutavani RV, Spendlove I, Ramage JM (2016). Stromal fibroblasts support dendritic cells to maintain IL-23/Th17 responses after exposure to ionizing radiation. J Leukoc Biol.

[81] Zhou Y, Su Y, Zhu H, Wang X, Li X, Dai C (2019). Interleukin-23 receptor signaling mediates cancer dormancy and radioresistance in human esophageal squamous carcinoma cells via the Wnt/Notch pathway. J Mol Med (Berl).

[82] Ruhland MK, Loza AJ, Capietto A-H, Luo X, Knolhoff BL, Flanagan KC (2016). Stromal senescence establishes an immunosuppressive microenvironment that drives tumorigenesis. Nat Commun.

[83] Guan X, LaPak KM, Hennessey RC, Christina YY, Shakya R, Zhang J (2017). Stromal senescence by prolonged CDK4/6 inhibition potentiates tumor growth. Mol Cancer Res.

[84] Duda DG, Duyverman AMMJ, Kohno M, Snuderl M, Steller EJA, Fukumura D (2010). Malignant cells facilitate lung metastasis by bringing their own soil. Proc Natl Acad Sci USA.

[85] Zhou P, Xiao N, Wang J, Wang Z, Zheng S, Shan S (2017). SMC1A recruits tumor-associated-fibroblasts (TAFs) and promotes colorectal cancer metastasis. Cancer Lett.

[86] Aceto N, Bardia A, Miyamoto DT, Donaldson MC, Wittner BS, Spencer JA (2014). Circulating tumor cell clusters are oligoclonal precursors of breast cancer metastasis. Cell.

[87] Wintzell M, Hjerpe E, Lundqvist EA, Shoshan MC (2012). Protein markers of cancer-associated fibroblasts and tumor-initiating cells reveal subpopulations in freshly isolated ovarian cancer ascites. BMC Cancer.

[88] Mccarthy JB, Elashry D, Turley EA (2018). Hyaluronan, cancer-associated fibroblasts and the tumor microenvironment in malignant progression. Front Cell Dev Biol.

[89] Ao Z, Shah SH, Machlin L, Parajuli R, Miller P, Rawal S (2015). Identification of cancer associated fibroblasts in circulating blood from patients with metastatic breast cancer. Can Res.

[90] Hessmann E, Patzak M, Klein L, Chen N, Kari V, Ramu I (2018). Fibroblast drug scavenging increases intratumoural gemcitabine accumulation in murine pancreas cancer. Gut.

[91] Hauge A, Rofstad EK (2020). Antifibrotic therapy to normalize the tumor microenvironment. J Transl Med.

[92] Tianyi L, Linli Z, Danni L, Thomas A, Zhang Y (2019). Cancer-associated fibroblasts build and secure the tumor microenvironment. Front Cell Dev Biol.

[93] Sahai E, Astsaturov I, Cukierman E, DeNardo DG, Egeblad M, Evans RM (2020). A framework for advancing our understanding of cancer-associated fibroblasts. Nat Rev Cancer.

[94] Hayden A, Manousopoulou A, Cowie A, Walker R, Sharpe B, Harrington J, et al. Modulation of the tumour promoting functions of cancer associated fibroblasts by phosphodiesterase type 5 inhibition increases the efficacy of chemotherapy in human preclinical models of esophageal adenocarcinoma. 2020;18:1249

[95] Broekgaarden M, Anbil S, Bulin AL, Obaid G, Mai Z, Baglo Y (2019). Modulation of redox metabolism negates cancer-associated fibroblasts-induced treatment resistance in a heterotypic 3D culture platform of pancreatic cancer. Biomaterials.

[96] Kirk D, Szalay MF, Kaighn ME (1981). Modulation of growth of a human prostatic cancer cell line (PC-3) in agar culture by normal human lung fibroblasts. Can Res.

